# First human implant of the cardiac contractility modulation in patient with dilated cardiomyopathy–related laminopathy

**DOI:** 10.1016/j.hrcr.2023.03.011

**Published:** 2023-03-24

**Authors:** Antonio D’Onofrio, Giuseppe Palmiero, Giuliano D’Alterio, Stefano De Vivo, Benedicta Maione, Silvia Leonardi

**Affiliations:** ∗ Electrophysiology and Cardiac Pacing Unit, AO dei Colli - Monaldi Hospital, Naples, Italy; † Inherited and Rare Cardiovascular Disease Unit, AO dei Colli - Monaldi Hospital, Naples, Italy; ‡ Clinical Biochemistry Unit, AO dei Colli - Monaldi Hospital, Naples, Italy

**Keywords:** Heart failure, Cardiomyopathy, Laminopathy, Cardiac contractility modulation, Electrical heart failure treatment

## Introduction

**Key Teaching Points**

• Cardiac contractility modulation can be used as a therapeutic aid for class III systolic heart failure patients with ejection fractions between 25% and 45%, not indicated for biventricular pacing.
• Dilated cardiomyopathy–related laminopathy is an inherited cardiomyopathy characterized by an ominous clinical course with life-threatening arrhythmias and heart failure.
• Cardiac contractility modulation could be a novel treatment in the prevention and/or delay of heart failure progression in patients suffering from dilated cardiomyopathy–related laminopathy.

Dilated cardiomyopathy (DCM)-related laminopathy (LMNA) is an inherited cardiomyopathy characterized by a higher rate of life-threatening arrhythmias and heart failure (HF) with respect to other heart diseases.^1^^,^^2^ LMNA mutations are found in 6% of all DCM cases and are associated with potential biventricular enlargement and systolic dysfunction. HF becomes apparent 15–20 years after the diagnosis and is associated with a high mortality rate (12%). The onset age of HF^3^^,^^4^ is around 30 in 10% of patients and 50 in 64% of patients.^5^

Currently, for LMNA-DCM^3^^,^^4^ patients are clinically managed by following standard protocols for HF, as there are no specific treatments for this condition.

In this case report, we investigate the application of cardiac contractility modulation (CCM) therapy in a patient with LMNA-DCM and chronic HF. The potential beneficial effects of CCM therapy have been evaluated on the following: (1) quality of life (QoL) improvement; (2) changes in left ventricular (LV) dimensions and volume, as well as consequent ejection fraction (LVEF) improvement; and (3) myocardial fibrosis development.

## Case report

A 48-year-old white man, suffering from chronic HF with reduced EF, was screened for LMNA gene mutation. His 50-year-old brother, with end-stage DCM, was identified as a proband. The patient has been followed up in our Electrophysiology Clinic since 2018 owing to first-degree atrioventricular block. Imaging evaluation, including transthoracic echocardiogram (TTE) and cardiac magnetic resonance, showed severe LV dysfunction (LVEF 28%) in the absence of significant coronary artery disease on diagnostic angiography. After 6 months of optimized medical treatment, the patient still presented moderate symptoms, such as shortness of breath (NYHA class II–III) and severely reduced exercise capacity (210 meters) on a 6-minute walking test. Moreover, no significant improvement in LVEF value (33%) was achieved.

Given his age, the patient was referred for a heart transplant and a dual-chamber defibrillator was implanted in 2019.

After 2 years, the patient was still symptomatic for HF despite a significant improvement in LVEF (40% vs 33% before implantable cardioverter-defibrillator [ICD] implantation) as a consequence of optimal medical therapy (OMT) including bisoprolol (2.5 mg twice daily), furosemide (25 mg twice daily), potassium-sparing diuretic (50 mg daily), and sacubitril/valsartan (49 mg sacubitril/51 mg valsartan twice daily). Dosage reinforcement was not possible owing to blood pressure and heart rate values, which were 90/60 mm Hg and 50–60 beats/min, respectively.

On March 2021, the patient was admitted once more with acute decompensated HF. The Kansas City Cardiomyopathy Questionnaire score and NT-proBNP value were 75.5 and 1520 pg/mL, respectively. The electrocardiogram showed sinus rhythm with first-degree atrioventricular block (390 ms), an average heart rate of 54 beats/min, and QRS =100 ms (Supplemental Figure 1). ICD interrogation showed 3% ventricular pacing since the implant, thanks to the algorithm for ventricular pacing prevention. No atrial arrhythmia events were recorded by the ICD. TTE showed a nondilated LV (LV end-diastolic volume 133 mL, LV end-systolic volume 80 mL) with moderate systolic dysfunction (LVEF 40% [Supplemental Videos 1 and 2], global longitudinal strain -11.3%, and myocardial work efficiency of 85%).

### Electrophysiology management

**Figure 1:**
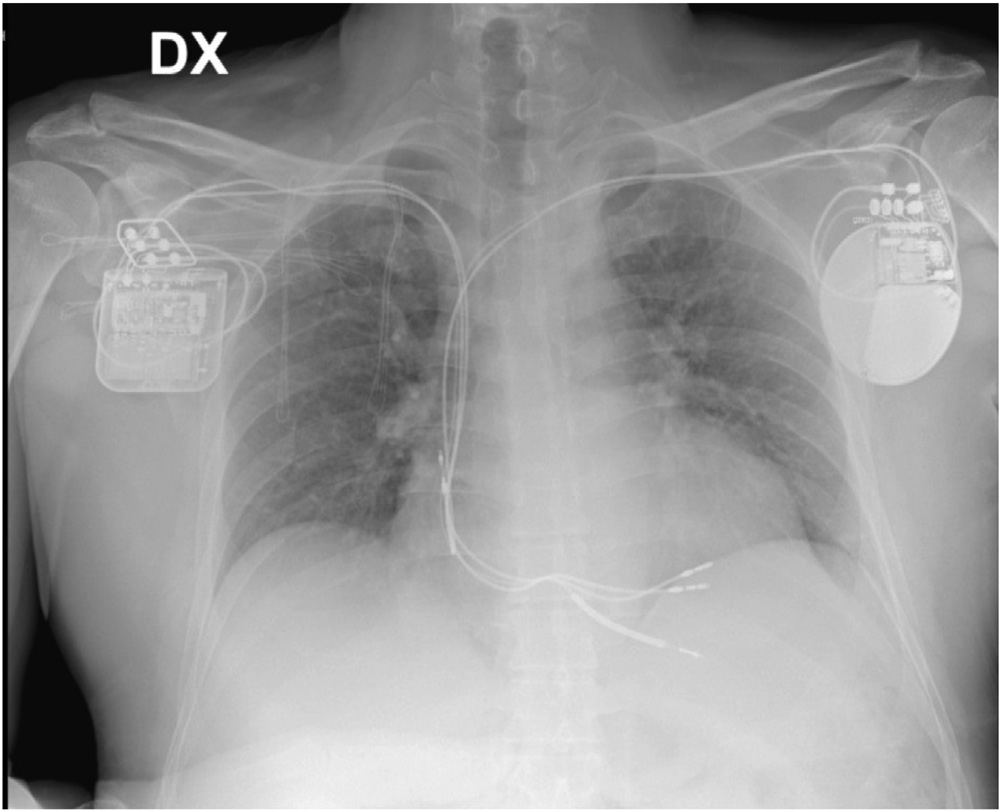
Chest radiograph of the patient. The figure shows cardiac contractility modulation (CCM) device on the right side and implantable cardioverter-defibrillator (ICD) DR on the left side. DX indicates right site; DR indicates a dual chamber device. Adx lead = right atrial lead for ICD; CCM1-RV = lead reference ventricular CCM; CCM2-LS = lead local sense CCM; ICD lead = right ventricular lead for ICD.

In the ESC HF 2019 consensus document,^6^ CCM therapy may be considered in symptomatic NYHA class III HF patients with narrow QRS and LVEF between 25% and 45%. Since the patient was still symptomatic for HF despite the use of intravenous diuretics and inotropes, a CCM device was implanted in March 2021, after collecting the signed informed consent, to improve symptoms and quality of life as well as prevent the worsening of the cardiomyopathy (Figure 1).

Therapy was scheduled for 8 hours per day, with the delivery of CCM therapy without any interference with the ICD previously implanted (Supplemental Figures 2 and 3).

Despite the implantation of the 2 additional leads in the right heart chamber for the delivery of CCM therapy, there was no increase in tricuspid regurgitation.

At the baseline evaluation, blood samples were collected to evaluate biomarkers of vascular inflammation (interleukin-6, phospholipase lipoprotein A2), fibrosis (laminin, type 3 and 4 collagen), HF (NT-proBNP, copeptin), and renal function (C-cystatin).

At 6 months follow-up, the patient showed improvements in clinical symptoms, QoL, laboratory biomarkers, and LV systolic function parameters on TTE, as exhibited in Table 1.

**Table 1:** Data collected at baseline, 6 months follow-up, and 12 months follow-up

Parameter	Baseline	6 months	12 months	Normal values
KCCQ	75.5	87.3	93.6	75–100
LVEDV [mL]	133	81	125	106 ± 22
LVESV [mL]	80	50	64	41 ± 10
LVEF [%]	40	50	50	62 ± 5
GLS [%]	-11.3	-13	-12.8	-18 ± 2
RVD1 [mm]	46	39	39	33 ± 4
RVD2 [mm]	42	42	42	27 ± 4
RVD3 [mm]	75	69	69	71 ± 6
NT-proBNP [pg/mL]	1520	801	675	0–125
Laminin [ng/mL]	34	28.13	13	0–50
Collagen 4 [ng/mL]	33	15.6	21	0–30
Collagen 3 [ng/mL]	18.1	16,5	21.3	0–30
Copeptin [pmol/L]	17.54	14.92	11.32	<17
C-cystatin [mg/L]	1.14	1.01	1.12	0.47–1.09
Lp-PLA2 [ng/mL]	1628	26.57	201.5	1–200
Interleukin-6 [pg/mL]	5.1	3.6	< 2	0–5

GLS = global longitudinal strain; KCCQ = Kansas City Cardiomyopathy Questionnaire.

**Figure 2:**
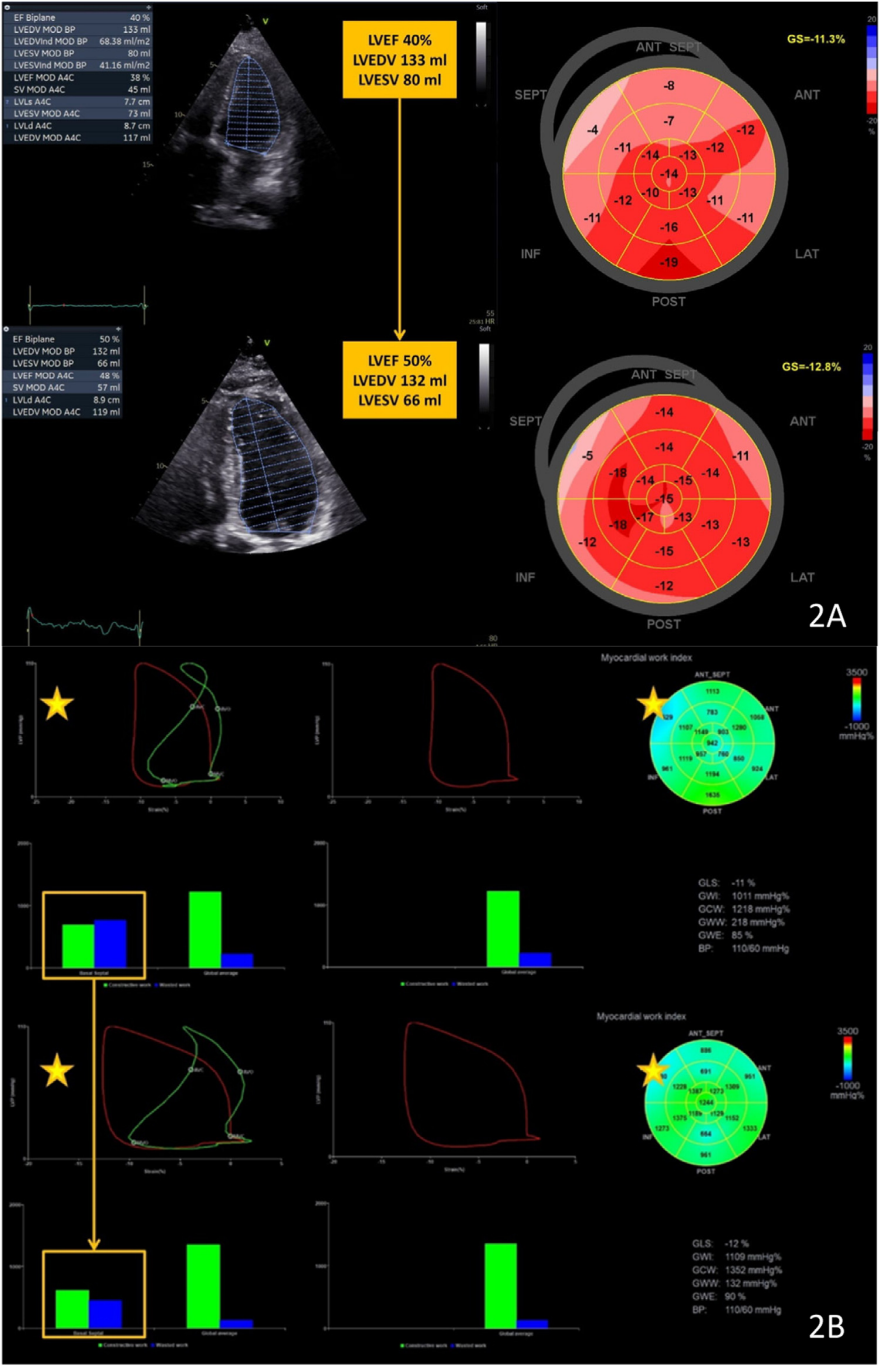
**A:** Comparison between baseline echo and echo at 12 months follow-up. **B:** Myocardial work analysis between baseline and 12 months follow-up. LVEDV = left ventricular end-diastolic volume; LVEF = left ventricular ejection fraction; LVESV = left ventricular end-systolic volume.

All improvements were confirmed at 12 months follow-up: QoL (NYHA class I, Kansas City Cardiomyopathy Questionnaire score 93.6) and LV systolic function parameters on TTE: LVEF 50% vs 40% (Supplemental Videos 3 and 4), global longitudinal strain -12.8 vs -11.3%, myocardial work efficiency 90% vs 85% (primarily driven by a significant reduction in global wasted work) (Figure 2).

Laboratory examinations showed a significant reduction in HF (NT-proBNP, copeptin) and inflammatory biomarkers (interleukin-6) in presence of stable values of fibrosis (laminin, type 3 and 4 collagen) and renal function markers (Table 1).

## Discussion

CCM therapy has been studied in a general population of NYHA class III patients with medically refractory therapy with LVEF 25%–45%, not indicated for biventricular pacing. Prospective randomized reports have demonstrated improvement in QoL, NYHA classification, 6-minute walking test distance, and peak oxygen consumption at cardiopulmonary exercise testing. Moreover, in this setting, CCM therapy significantly improved combined endpoint of cardiovascular death and HF hospitalization.^7^^,^^8^ Recent observations from the CCM European Registry described a potential benefit in mortality for those patients with LVEF between 35% and 45% range. These patients also experienced an average absolute increase of approximately 5% in the LVEF.^9^ The actual ESC HF Guidelines, published in August 2021, state that CCM therapy is under evaluation to reduce mortality and hospitalization. However, more actual clinical evidence is needed to give CCM a class of recommendation.^10^

The patient evaluated in this case report was on the heart transplant waiting list and refused the LV assist device implant. Furthermore, the OMT at the time of the implantation did not include the administration of SGLT-2 inhibitors. The CCM therapy treatment reverses the cardiac maladaptive fetal gene and normalizes the expression of key sarcoplasmic reticulum Ca2+ cycling and stretch response genes.^11^

The rationale of CCM therapy in LMNA-DCM patients is based on the analysis of data from human and animal models that suggest the inhibition of mitogen-activated protein kinase (MAPK) signaling and mTOR pathways, which is beneficial in preventing and treating cardiac dysfunction^4^ (Supplemental Figure 4). In a crossover study, Butter and colleagues^11^ found that the MAPK expression reduction after CCM therapy indicates a reduced mechanical stress. Regarding the role of MAPK in LMNA-DCM, data from a mouse model suggest that MAPK inhibitor signaling pathways are useful in preventing and treating cardiac dysfunction.^12^ AKT/mTOR pathway appears to be another sign of early-activated signaling in LMNA-DCM in animal models. AKT expression in cardiac myocytes could promote cardiac growth, myocardial angiogenesis, hypertrophy, hyperplasia, and cell apoptosis. A few studies are focused on the responses of the AKT pathway proteins to CCM in a rabbit model with HF. Specifically, the inhibition of these signaling pathways after CCM has shown encouraging beneficial effects on the cardiac evolution of LMNA-DCM.^13^ It is also reported that CCM could significantly alter cytoskeleton proteins and matrix metalloproteinases.^14^ Inhibition of the AKT/mTOR pathway is associated with a reduction of myocardial fibrosis in patients with HF and contributes to reverse cardiac remodeling.^14^ Several scientific publications indicate that the activity of these pathways is decreased after the application of the CCM therapy, which results in the decrease of cardiac fibrosis, collagen deposition, mechanical stresses, and improvement of the clinical effects.^14^^,^^12^

Our aim was to evaluate the effect of CCM therapy in patients with LMNA-DCM and symptomatic HF despite OMT with QRS less than 130 ms, NYHA class equal to or higher than II, and EF between 25% and 45%. It is necessary to have a large cohort of patients to confirm the application of CCM therapy in this condition and other familiar cardiomyopathies such as titin dystrophies, as shown in a recent case report by Hesselson and colleagues.^15^ The introduction into the market of a device combining an ICD with CCM therapy could help to treat this type of patient.

We established a CCM therapy registry on ClinicalTrials.gov (CARDILAM CCM registry, NCT 04904393) to investigate patients with LMNA-DCM and validate our approach.

## Conclusion

In our case report, we observed an improvement of QoL, echo parameters, and inflammatory biomarkers following CCM device implantation. The results of this case report suggest that CCM could be a possible novel treatment in patients with LMNA-DCM to prevent and/or delay the progression of HF. Further large-scale studies are needed to confirm this hypothesis.

## Figures and Tables

**Supplemental Video 1:**
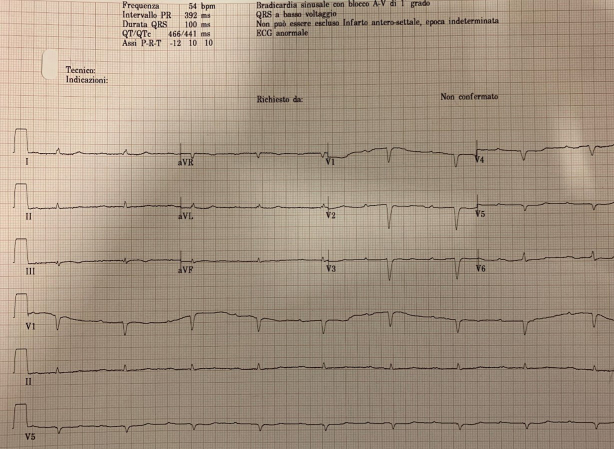
Echo Exam Baseline 2-Chamber view1

**Supplemental Video 2:**
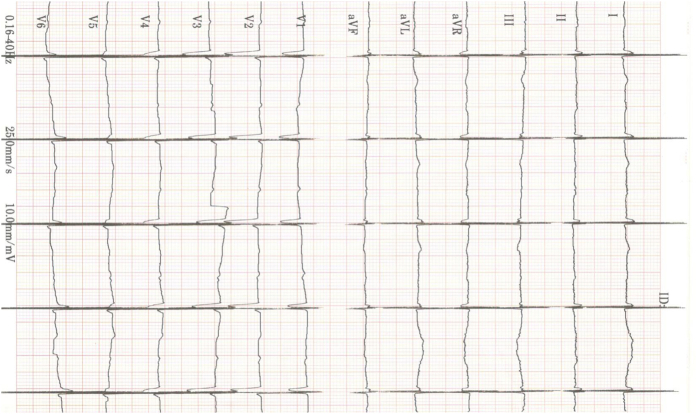
Echo Exam Baseline 4-Chamber view2

**Supplemental Video 3:**
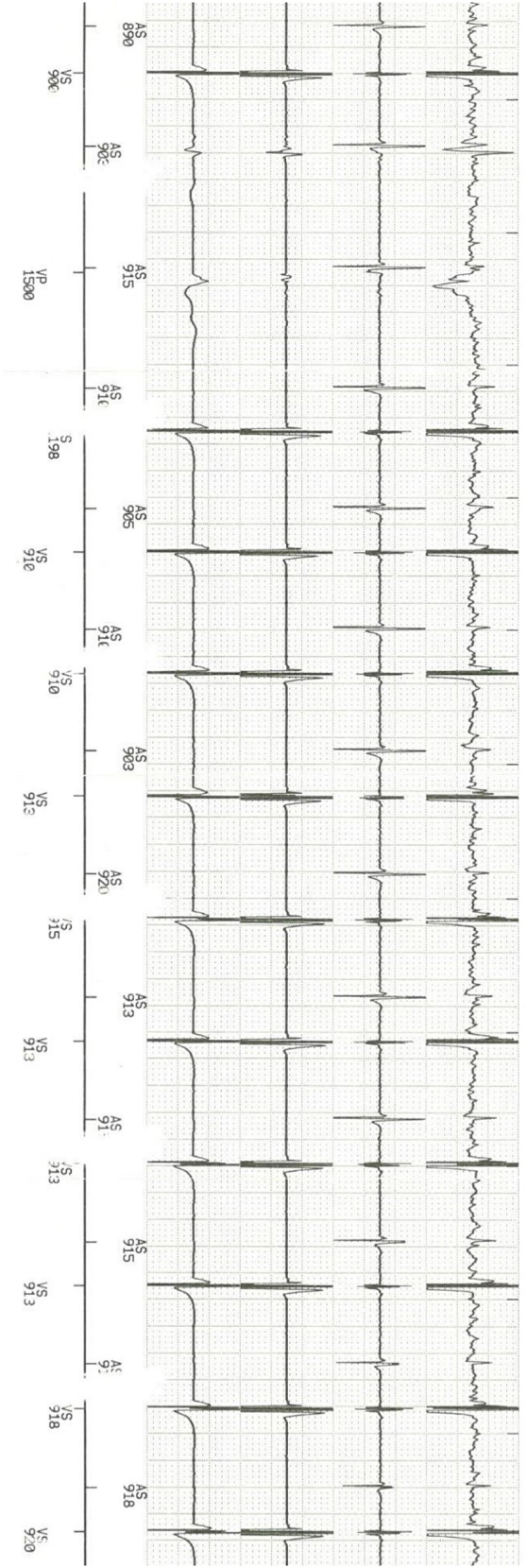
Echo Exam 12 months FU 2-Chamber view3

**Supplemental Video 4:**
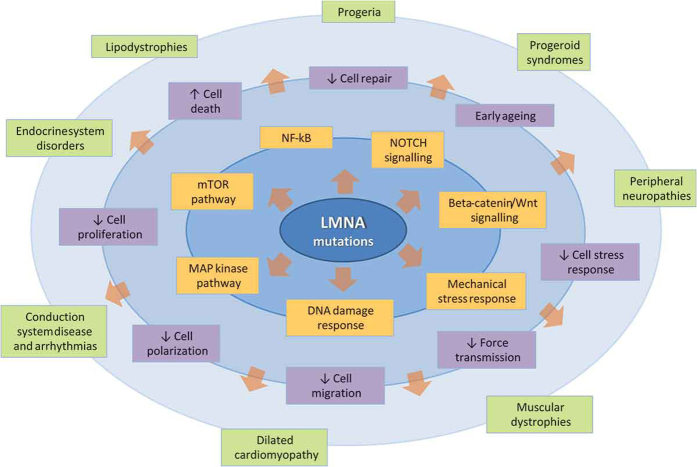
Echo Exam 12 months FU 4-Chamber view4

## Appendix Supplementary Data

Supplemental Figure 1 Baseline EKG showed sinus rhythm with I degree AV block (390 ms) and a mean heart rate of 54 beats-per-minute and QRS =100 ms

Supplemental Figure 2 Post implant EKG shows the CCM spike during ventricular refractory period

Supplemental Figure 3 IEGM from ICD shows no interference and no double counting between CCM spike and ICD atrial and ventricular channels

Supplemental Figure 4 Pathophysiological overview of laminopathies: the possible inhibition of mitogen-activated protein kinase (MAPK) signaling and mTOR pathways could be beneficial in term of prevention and treatment of cardiac dysfunction (Reference n 4 - Peretto G, Sala S et al. Updated clinical overview on cardiac laminopathies: an electrical and mechanical disease. Nucleus 2018, Vol 9, No 1, 380-391).
